# Conventional type 1 dendritic cells in the lymph nodes aggravate neuroinflammation after spinal cord injury by promoting CD8^+^ T cell expansion

**DOI:** 10.1186/s10020-024-01059-4

**Published:** 2025-02-03

**Authors:** Li-Qing Wang, Xiao-Yi Wang, Yue-Hui Ma, Heng-Jun Zhou

**Affiliations:** 1 Department of Anesthesiology, The First Affiliated Hospital, College of Medicine, Zhejiang University, Hangzhou, 310003 China; 2 Department of Neurosurgery, The First Affiliated Hospital, College of Medicine, Zhejiang University, 79 Qingchun Rd., Hangzhou, 310003 People’s Republic of China

**Keywords:** Spinal cord injury, Neuroinflammation, Conventional type 1 dendritic cells, Lymph nodes, CD8^+^ T cell

## Abstract

**Background:**

Adaptive immune response is at the core of the mechanism of secondary spinal cord injury (SCI). This study aims to explore the molecular mechanism by which classical dendritic cells (cDC1s) influence CD8^+^ T cell expansion in SCI.

**Methods:**

Peripheral blood samples from patients with SCI and spinal cord tissues from SCI mice were collected, and the population of cDC1 subset was analyzed by flow cytometry. In vivo, the fms-like tyrosine kinase 3 (Flt3) inhibitor quizartinib was administered to deplete cDC1s, while intraperitoneal injection of recombinant Flt3L and immunosuppressive drug FTY-720 was used to expand cDC1s and prevent T cell egress from lymph nodes (LNs), respectively. In vitro, the conditioned medium (CM) of isolated LN fibroblastic stromal cells (FSCs) and pre-DCs were co-cultured. Subsequently, FSC CM-induced DCs were stimulated and co-cultured with CD8^+^ T cells for proliferation assay.

**Results:**

The cDC1 subset was increased in the peripheral blood of SCI patients and in the injured spinal cord of SCI mice. Depletion of cDC1s decreased the proportion of infiltrating CD8^+^ T cells in the injured spinal cord of SCI mice and reduced the inflammatory response. The Basso Mouse Scale score of SCI mice was increased and the proportion of CD8^+^ T cells in blood and spinal cord tissue was decreased after FTY-720 injection. Both migratory cDC1s (CD103^+^) and resident cDC1s (CD8α^+^) were present in the LNs surrounding the injured spinal cord of SCI mice. Among them, CD103^+^ cells were derived from the migration of cDC1s in spinal cord tissues, and CD8α^+^ cDC1s were directionally differentiated from pre-DCs after co-culture with LN-FSCs. Interferon-γ promoted the secretion of Flt3L by LN-FSCs through the activation of JAK/STAT signaling pathway and enhanced the differentiation of pre-DCs into CD8α^+^ cells.

**Conclusion:**

Migratory cDC1s and resident cDC1s promote the expansion of CD8^+^ T cells in LNs around the injured spinal cord and mediate the adaptive immune response to aggravate neuroinflammation in SCI.

**Supplementary Information:**

The online version contains supplementary material available at 10.1186/s10020-024-01059-4.

## Introduction

Spinal cord injury (SCI) is a type of severe traumatic disease of the central nervous system (CNS), and the treatment of SCI is also one of the major problems facing the medical community at present (Badhiwala et al. 2019). The pathophysiological changes after SCI can be divided into primary injury and secondary injury. Primary injury is an irreversible mechanical injury caused by an external cause (car accident, fall from height, or violent fracture, *etc*.) or an internal cause (myelitis or spinal cord tumor, *etc*.) that acts directly on the spinal cord (Ahuja et al. 2017). Secondary damage refers to a series of molecular damages caused by indirect causes that severely aggravate the damage caused by the primary damage (Anjum et al. 2020). Due to the unpredictability and uncontrollability of primary injury, scientists began to pay attention to the series of changes caused by secondary SCI. The excessive immune response of the body will lead to secondary damage, which will affect the recovery of nerve function (Sterner and Sterner 2022). Therefore, regulating the adaptive immune response to reduce secondary SCI is an effective way to improve the therapeutic effect of SCI (Cadotte and Fehlings 2011).

T and B lymphocytes are an important part of the adaptive immune response, of which CD8^+^ T cells are the major cytotoxic cells (Taniuchi 2018; Harkins et al. 2023). In peripheral lymphoid tissues, antigen-specific naive CD8^+^ T cells are activated after encountering homologous antigens presented by dendritic cells (DCs) and are rapidly cloned to produce large numbers of antigen-specific effector CD8^+^ T cells that enter the circulation and migrate to infection or tumor, secrete proinflammatory cytokines [interferon-γ, tumor necrosis factor-α, cytotoxic effector molecules (perforin, granzyme, *etc.*)], and specifically eliminate infected cells or tumor cells (Harty and Badovinac 2008). After the onset of SCI, microglia are activated, monocyte/macrophage cells and CD4^+^ T cells, CD8^+^ T cells, B cells involved in adaptive immunity are also infiltrated (Erens et al. 2022). Wang et al*.* found that activated CD8^+^ T cells increased in the injured spinal cord and inhibited the growth of neural stem cells after SCI, and intraperitoneal injection of anti-CD8 antibody to deplete CD8^+^ T cells helped to promote the recovery of motor function in mice with SCI (Wang et al. 2023). In addition, Liu et al. found that perforin secreted by CD8^+^ T cells aggravated secondary SCI by disrupting the blood-spinal cord barrier (Liu et al. 2019). Therefore, we hypothesized that inhibition of adaptive immunity involving CD8^+^ T cells could alleviate secondary SCI.

DCs are derived from CD34^+^ hematopoietic stem cells in the bone marrow, where hematopoietic stem cells produce common myeloid and lymphoid precursors that are stimulated to differentiate into classical DCs (cDCs) and plasma cell-like DCs, respectively (Villar and Segura 2020). cDCs are important cells that mediate the adaptive immune response, which are divided into cDC1s and cDC2s according to the nature of the antigen and the difference in activated T lymphocytes (Anselmi et al. 2020). By recognizing danger signals released by the body, cDC1s differentiate and mature to take up antigens and present them to CD8^+^ T cells (Freeman and Curtis 2017). Traumatic brain injury (TBI) and SCI are both central nervous system traumas, and studies have shown that cDC1s are significantly increased in spleen, blood, and mesenteric lymph nodes (LNs) after TBI (Hu et al. 2020). In summary, we aimed to investigate whether cDC1s mediate the adaptive immune response by regulating the expansion of CD8^+^ T cells in SCI.

## Materials and methods

### Clinical information

A total of 10 patients with SCI who were treated at the First Affiliated Hospital, Zhejiang University School of Medicine from January 2023 to September 2023 were selected as study subjects. The inclusion criteria were as follows: meeting the international standard for classification of nerve function in spinal cord injury; over 18 years of age; no clear history of stroke or other diseases that may affect brain structure and cognitive function; patients with Montreal Cognitive Assessment Scale < 26; clear consciousness, understanding, communication ability, and voluntary participation in the study. Exclusion criteria were as follows: American Spinal Injury Association (ASIA) Grade A neurological function classification; have other serious physical or neurological disorders. Ten healthy cases with healthy physical examination and matched for sex and age in the same period were used as healthy control. This study was approved by the ethics committee of the First Affiliated Hospital, Zhejiang University School of Medicine. All subjects participated voluntarily and signed an informed consent form. 5 mL of peripheral blood (stored in tubes containing EDTA anticoagulant, Becton Dickinson) was collected and centrifuged at 1000 rpm for 10 min. The lower blood cells were collected using human lymphocyte separation medium (Beijing Solarbio Science & Technology Co., Ltd.), peripheral blood mononuclear cells (PBMCs) were separated by density gradient centrifugation method and frozen at -80℃ for later use.

### Establishment of the SCI mouse model

Female C57BL/6 mice aged 6 to 8 weeks (weight 20–25 g) were purchased from Zhejiang Vital River Laboratory Animal Technology Co., Ltd. The model of acute SCI was established according to our previous method (Zhou et al. 2022). Briefly, mice were anesthetized by intraperitoneal injection of 1% pentobarbital sodium (60 mg/kg), the 9th thoracic vertebral lamina was removed, and the spinal cord was fully exposed. After the spine was stabilized, the SCI model was established by vertically impacting the spinal cord with a 50 kDynes animal SCI impactor (diameter 1.3 mm, IH-0400, PSI, USA). The mice showed spasm and tail wagging and paralysis of both lower limbs after the impact, indicating that the modeling was successful. In the sham group, only the laminae were removed without damaging the spinal cord. After modeling, the mice were injected subcutaneously daily with 0.5 mL of 8000 U penicillin to prevent infection, and the bladders were manually emptied 3 times daily until the mice urinated autonomously. This study was approved by the Animal Experimental Ethics Committee of the First Affiliated Hospital, School of Medicine, Zhejiang University.

### Drug administration and grouping

In the SCI + Qu group, SCI mice were treated with the fms-like tyrosine kinase 3 (Flt3) inhibitor quizartinib (Qu, 10 mg/kg, Selleckchem, USA) by oral gavage daily starting on day 14 after surgery (Chen et al. 2023), and SCI group mice were treated with phosphate-buffered saline (PBS) as a control. In the SCI + Flt3L group, Flt3 ligand (Flt3L, 30 μg in 100 μL PBS, Peprotech) was injected intraperitoneally daily starting at 7 days post injury (dpi). In the SCI + FTY-720 group, FTY-720 (40 μg in 100 μL saline, Sigma) was injected intraperitoneally every 3 days starting at 14 dpi. In the SCI + Flt3L + FTY-720 group, Flt3L and FTY-720 were injected intraperitoneally until 42 dpi. There were 6 mice per group.

### Detection of basso mouse scale (BMS) scores

The BMS scores were used to evaluate the recovery of motor function in the sham and SCI groups. The continuous motor status of the hind limbs of the mice was assessed before surgery and on days 3, 7, 14, 21, 28, 35, and 42 after surgery (Chen et al. 2021). Mice were placed in an open flat field and allowed to move freely for 3 min. The evaluation was performed in a double-blind fashion. The observation indices included ankle movement of the mice's hind limbs, coordination, paw posture, and trunk stability, *etc*.

### Immunofluorescence (IF) detection

After determination of the BMS score, spinal cord tissues were harvested, paraffin embedded (3 μm), deparaffinized, dewaxed, and antigen retrieved in sequence. Subsequently, 0.3% bovine serum protein was added and sections were incubated at room temperature and blocked for 30 min. After incubation with primary antibodies at 4 ℃ overnight, sections were incubated at 4 ℃ overnight, the second antibody was added, and then incubated at 37 ℃ for 30 min. Then 4',6-diamidino-2-phenylindole (DAPI) fluorescence dye solution was added and incubated for 5 min. The film was sealed and observed under a fluorescence microscope (Olympus, Japan). Primary antibodies were as follows: anti-CD11C (MA11C5, eBioscience), anti-major histocompatibility complex II (MHC-II, ab23990, Abcam), anti-chemokine (C motif) receptor 1 (XCR1, A04185-1, Boster), anti-CD8α (ab217344, Abcam), anti-lymphatic vessel endothelial hyaluronan receptor 1 (LYVE-1, ab281587, Abcam).

### Sample processing and flow cytometry

The proportion of cDC1s (CD141^+^) and cDC2s (CD1c^+^) of cDCs (CD11C^+^HLA-DR^+^) in PBMCs was determined by flow cytometry (Schlitzer et al. 2015). The PBMCs obtained by the above method were washed twice with phosphate buffer (PBS). After discarding the supernatant, cells suspended in PBS were added, the cell concentration was adjusted to 5 × 10^7^ cells/mL, and 200 μL of the cell suspension was absorbed into the tube. Flow cytometry antibodies were added and incubated for 30 min in the dark. Then, 3 mL PBS was added for washing, centrifuged at 1000 rpm for 5 min, and the supernatant was discarded. After the addition of 300 μL PBS to the suspended cells, APC-conjugated CD11C (#980604, Biolegend), PE-conjugated HLA-DR (#327007, Biolegend), FITC-conjugated CD1c (#331517, Biolegend), FITC-conjugated CD141 (#11-1419-42, eBioscience) were used to detect the proportion of CD141^+^CD11C^+^HLA-DR^+^ or CD1c^+^CD11C^+^HLA-DR^+^ cell subsets by flow cytometry (FAC-S Calibur, BD).

Spinal cord tissues of 8 mm length were collected from the injured area and digested with artificial cerebrospinal fluid containing 1 mg/mL collagenaseII (Gibco) and 1 mg/mL papain (Gibco) at 37 ℃ for 30 min. After trituration, the suspension was washed twice with fluorescence-activated cell sorting (FACS) buffer. Isolated single cells were collected after density gradient centrifugation and red blood cells were removed. The leukocytes were then collected and washed twice with FACS buffer for flow cytometry. APC-conjugated CD3 (#10235, Biolegend), FITC-conjugated CD4 (#100510, Biolegend), and PE-conjugated CD8 (#100707, BioLegend) were used to screen for CD8^+^ T cell subsets in mouse spinal cord tissue. CD45^+^ cells were sorted using CD45 magnetic beads (#8802-6865-74, ThermoFisher), and then XCR1 (percp, 148207, Biolegend) and FITC-conjugated CD103 (#110907, Biolegend) were used to detect the proportion of cDC1s (XCR1^+^CD103^+^CD45^+^) in mouse spinal cord tissue.

Peripheral blood was collected from mouse eyes at 42 dpi and anticoagulated with EDTA. After erythrocyte lysis, leukocyte surface markers were isolated and analyzed by flow cytometry (Deczkowska et al. 2021). APC-conjugated CD3 (#100235, Biolegend), FITC-conjugated CD4 (#100510, Biolegend) and PE-conjugated CD8 (#100707, BioLegend) were used to screen CD8^+^ T cell subsets in mouse blood. PE-conjugated CD11C (#557401, Biolegend), I-A/I-E (Alexa Fluor® 647, #562367, BD biosciences), and FITC-conjugated CD103 (#110907, Biolegend), or FITC-conjugated CD11b (#101205, Biolegend) were used to detect the proportion of cDC1s (CD11C^+^I-A/I-E^+^CD103^+^) or cDC2s (CD11C^+^I-A/I-E^+^CD11b^+^) in mouse blood.

Deep cervical lymph nodes (dcLN) and lumbar lymph nodes (LLN) were collected and digested in Isocove's modified Dulbecco's medium supplemented with 100 μg/mL Liberase TL (Roche) and 100 μg/mL DNaseI (ThermoFisher) at 37℃ for 20 min. Leukocytes were then harvested and used for flow cytometry. PE-conjugated CD11C (#557401, Biolegend), I-A/I-E (Alexa Fluor® 647, #562367, BD biosciences), and FITC-conjugated CD103 (#110907, Biolegend) or FITC-conjugated CD8α (#100705, Biolegend) were used to detect the proportion of migratory cDC1s (CD11C^+^I-A/I-E^+^CD103^+^) or resident cDC1s (CD11C^+^I-A/I-E^+^CD8α^+^) in the dcLN or LLN of mice. PE-conjugated CD11C (#557401, Biolegend), I-A/I-E (Alexa Fluor® 647, #562367, BD biosciences) and XCR1 (percp, #148207, Biolegend) were used to detect the proportion of cDC1s (CD11C^+^I-A/I-E^+^XCR1^+^) in the dcLN or LLN of mice. PC-conjugated CD3 (#100235, Biolegend), PE-conjugated CD8 (#100707, Biolegend) and Ki67 (Alexa Fluor® 488, ab281847, Abcam) were used to detect the proportion of proliferating CD8^+^ T cells (CD3^+^CD8^+^Ki67^+^) T cells in mouse dcLN or LLN.

Isolated bone marrow was subjected to erythrocyte lysis and leukocytes were used for flow cytometry. FITC-conjugated CD45 (#157607, Biolegend), PE-conjugated CD11C (#557401, Biolegend), and APC-conjugated CD135 (#135309, Biolegend) were used to detect leukocytes (CD45^+^) and the proportion of pre-DCs (CD135^+^CD11c^+^CD45^+^) in mouse bone marrow.

### Enzyme linked immunosorbent assay (ELISA)

After weighing, the spinal cord tissues were sectioned, PBS was added at 1:9 (M/V), and the homogenate was completely ground on ice. The homogenate was centrifuged at 4 ℃, 12000 rpm for 10 min. The culture medium of LN fibroblastic stromal cells (FSCs) in each group was collected and centrifuged at 12000 rpm. The supernatant was collected, and the levels of interferon-γ (IFN-γ) and Flt3L were detected according to the instructions of the ELISA kit (ThermoFisher).

### Isolation of LN-FSCs, pre-DCs and CD8^+^ T cells

Isolated mouse LNs were digested with 5 mL of mixed digestion enzyme (0.2 mg/mL collagenaseII, 0.1 mg/mL DNaseI, and 0.8 mg/mL DispaseII) at 37 °C for 30 min, during which time they were repeatedly aspirated with a pipette. After digestion was stopped by adding RPMI 1640 medium, the cell suspension was filtered through a 40-μm nylon filter. The filtrate was collected and centrifuged at 1000 rpm for 8 min. The cell precipitates were then resuspended in RPMI 1640 medium containing 10% FBS (Gibco) and 1% penicillin–streptomycin and cultured at 37 °C in a 5% CO_2_ incubator. The next day, the medium was changed to remove suspended cells, and adherent LN-FSCs were cultured for another 5 days. In the sham FSC + IFN-γ group, 20 ng/mL IFN-γ was added and cultured for 5 days. In the SCI FSC + Tofa group, 1 μM tofacitinib (Tofa) was added and cultured for 5 days.

Mouse femurs and tibias were removed and placed in a dish containing 4 mL of RPMI 1640 medium supplemented with 10% FBS. The anterior and posterior ends of the femur were cut off to expose the cross section. A 1 mL syringe containing PBS was inserted into the bone marrow from the cross section, and the cells were washed repeatedly. After the addition of lysis buffer (Merck) to lyse red blood cells, the cells were resuspended in PBS after centrifugation and enriched using CD45 MicroBeads (#30-F11, Miltenyi Biotec). FITC-conjugated CD45 (#157607, Biolegend), PE-conjugated CD11C (#557401, Biolegend), and APC-conjugated CD135 (#135309, Biolegend) were used to sort pre-DCs.

Single cell suspensions of mouse LNs were subjected to erythrocyte lysis. The cells were then washed in PBS and CD8^+^ T cells were enriched with CD8 MicroBeads (#130-045-201, Miltenyi Biotec). CD8^+^ T cells were identified by flow cytometry.

### Western blot assay

The dcLN, LLN, and spinal cord tissues from sham and SCI group mice were collected, and the protein lysate was added for complete lysis. After standing and centrifugation, the supernatant was extracted. When cell confluence reached 80%, LN-FSCs from each group were collected and total protein was extracted. The protein concentration was measured according to the instructions of the BCA protein concentration assay kit (ThermoFisher), and then 5 × loading buffer was added according to the ratio, mixed thoroughly, and boiled at 100 ℃ for 10 min before use. The protein expression levels of Flt3L, IFN-γ, total signal transducers and activators of transcription (Stat) 1/3/5 (t-Stat1/3/5), phosphorylated Stat1/3/5 (p-Stat1/3/5) of different groups were compared after sample loading, electrophoresis, membrane transfer, blocking antibody, primary antibody incubation, secondary antibody incubation, visualization, and band analysis. The primary antibodies were as follows: Flt3L (ab52648, 1:100000, abcam), IFN-γ (sc-373727, 1:200, Santa Cruz), t-Stat1 (sc-464, 1:300, Santa Cruz), t-Stat3 (sc-8019, 1: 300, Santa Cruz), t-Stat5, p-Stat1 (sc-8394, 1:400, Santa Cruz), p-Stat3 (sc-8059, 1:400, Santa Cruz), p-Stat5, β-actin (sc-8432, 1:500, Santa Cruz).

### cDC1 differentiation analysis

The supernatant of LN-FSCs cultured for 24 h was collected as conditioned medium (CM). Pre-DCs sorted by flow cytometry were seeded into 96-well plates (3 × 10^3^ cells/mL) with 200 μL sham FSC CM or SCI FSC CM. After 3 days, fresh medium was replaced and cells were incubated for another 5 days at 37 °C in 5% CO_2_. For Flt3L blocking, Flt3L blocking antibody was added to FSC CM at 0 and 3 d at 5 μg/mL, while IgG was used as isotype control.

After co-culturing pre-DCs with FSC CM, cells in each group were washed twice with PBS and centrifuged at 2000 rpm for 5 min. PE-conjugated CD11C (#557401, Biolegend), I-A/I-E (Alexa Fluor® 647, #562367, BD biosciences) and FITC-conjugated CD8α (#100705, Biolegend) were used to detect the cDC subset and the ratio of resident cDC1s (CD11C^+^I-A/I-E^+^CD8α^+^).

### CD8^+^ T cell proliferation assay

FSC CM-induced DCs were stimulated with 1 μg/mL ovalbumin (OVA257-264) peptide for 2 h. Then, DCs (1 × 10^4^ cells/mL) were co-cultured with carboxycein diacetate succinimidyl ester (CFSE, Invitrogen)-labeled CD8^+^ T cells (1 × 10^5^ cells/mL) in 96-well plates at 100 μL with RPMI 1640 culture medium for 4 days. Cells were harvested and CD8^+^ T proliferation was assessed by flow cytometry.

### Statistical analysis

Data included in this study were measured mean ± standard deviation and analyzed using Graphpad software. One-way ANOVA with Tukey's post hoc or two-way ANOVA was used to compare data between multiple groups, and unpaired two-tailed T test or Mann–Whitney U test was used to compare data between two groups. *P* < 0.05 was considered statistically significant.

## Results

### cDC1s are elevated in patients with SCI and in mouse models of SCI

**Fig. 1:**
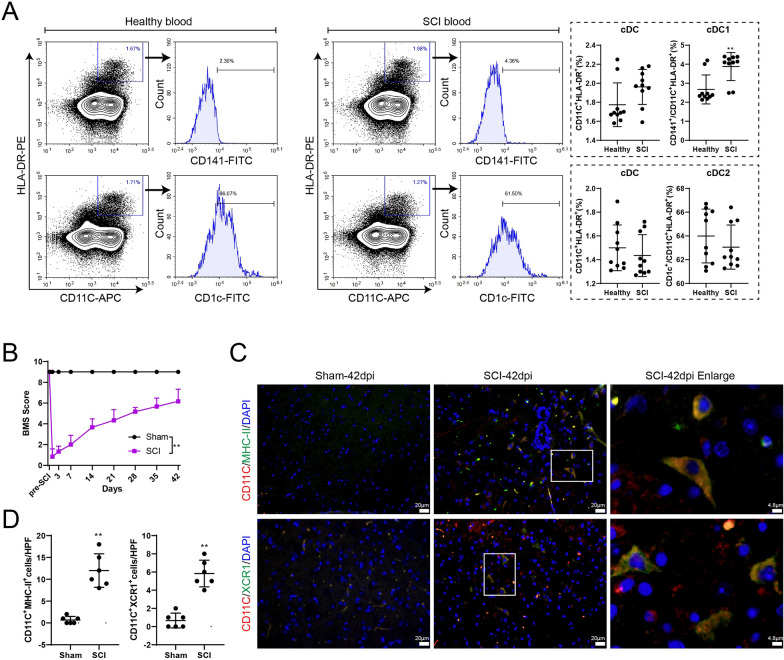
cDC1s in SCI patients and mouse model of SCI. **A** PBMCs were collected from 10 healthy volunteers and 10 SCI patients. Identification of cDC1s and cDC2s subsets was performed by flow cytometry. CD11C^+^HLA-DR^+^ represents cDCs, while CD141^+^ represents sDC1s and CD1c^+^ represents cDC2 cells. Difference was calculated by Mann–Whitney U test. **P < 0.01 *vs.* healthy group. **B** Mice were divided into sham (*n* = 6) and SCI (*n* = 6) groups. BMS score detection was performed. Difference was calculated by two-way ANOVA test. **P < 0.01 *vs.* sham group. **C–D** The positive expressions of MHCII and XCR1 in spinal cord tissues were detected by IF assay. The difference was calculated by unpaired two-tailed T-test. **P < 0.01 *vs.* sham group. *SCI*: spinal cord injury; *dpi*: days post-injury; *cDC*: conventional dendritic cell

To investigate the changes of cDCs after SCI, we collected blood from 10 healthy volunteers and 10 SCI patients, and then isolated PBMCs. The results of flow cytometry detection showed that cDC1s (CD11C^+^HLA-DR^+^CD141^+^) were significantly increased in the SCI blood group, but the proportion of cDC2s (CD11C^+^HLA-DR^+^CD1c^+^) was not significantly changed (Fig. 1A). Next, the SCI mouse model was successfully established. Compared with the sham mouse group, the BMS score of the SCI mouse group was significantly lower, indicating that the motor function of the mice was impaired after SCI (Fig. 1B). XCR1 and MHCII are specific markers on the surface of cDC1s. The IF staining results in Fig. 1C–D showed that infiltrated CD11C^+^MHCII^+^ and CD11C^+^XCR1^+^ cells near the injured spinal cord were increased, indicating an increase in cDC1s content after SCI.

### cDC1 depletion reduces CD8^+^ T cell infiltration after SCI injury

**Fig. 2:**
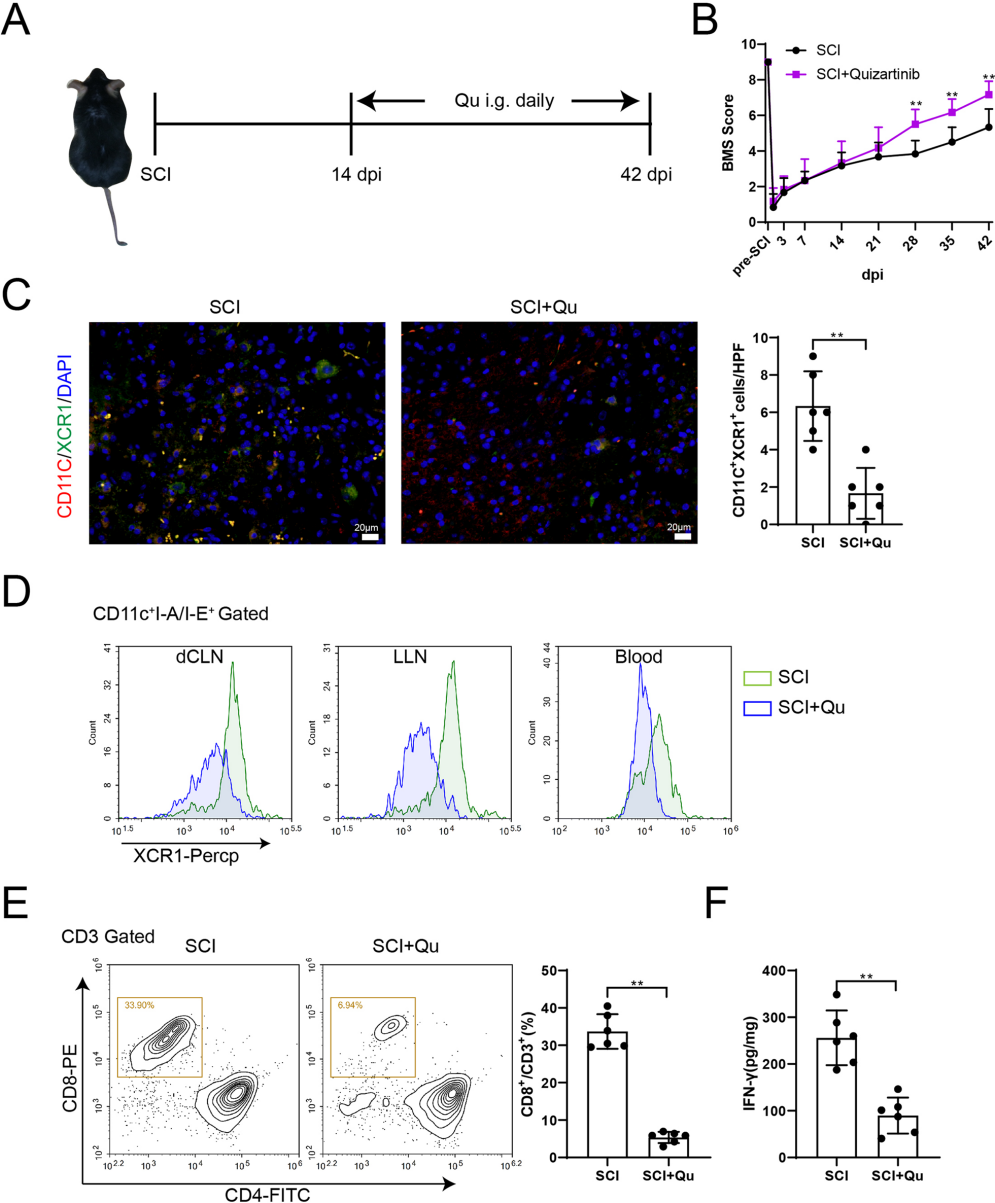
cDC1 depletion reduces CD8^+^ T cell infiltration after SCI injury. **A** Mice were divided into SCI (*n* = 6) and SCI + Qu (*n* = 6) groups. Qu administration was started on day 14 after surgery. **B** BMS score was determined. The difference was calculated by unpaired two-tailed T-test. **P < 0.01 *vs.* SCI group. **C** Positive expression of XCR1 in spinal cord tissues was detected by IF assay. The difference was calculated by unpaired two-tailed T-test. **P < 0.01. **D** The results of flow cytometry of XCR1^+^ cDCs in lymph nodes and blood. The cDC population was expressed as CD11C and I-A/I-E (MHCII) double positive, and XCR1^+^ represents XCR1^+^ cDCs. **E** The proportion of CD8^+^ T cells in spinal cord tissues was determined by flow cytometry. CD3^+^ T cells were circled with CD3 gate, and further CD3^+^CD8^+^ T cells were analyzed. The difference was calculated by unpaired two-tailed T-test. **P < 0.01 *vs.* SCI group. *N* = 6. **F** ELISA was used to detect IFN-γ levels in spinal cord tissues. The difference was calculated by unpaired two-tailed T-test. **P < 0.01 *vs.* SCI group. *N* = 6. *dpi*: days post-injury; *Qu*: Quizartinib

Flt3L is an important regulator of DC proliferation and differentiation (Wilson et al. 2021). To investigate whether cDC1s affect the infiltration of CD8^+^ T cells, SCI mice were orally gavaged with Flt3 inhibitor (Qu) daily starting from 14 dpi to deplete cDC1s (Fig. 2A). Figure 2B showed the BMS scores of SCI and SCI + Qu groups mice from 0 to 42 days, the results showed that the motor function of mice was restored after 28 dpi. In addition, the reduction of cDC1s in the injured spinal cord of SCI + Qu group mice was observed (Fig. 2C). The results of flow cytometry of XCR1^+^ cDCs (CD11C^+^I-A/I-E^+^XCR1^+^) in lymph nodes as well as in blood were shown in Fig. 2D and S2A, further indicating that Qu treatment was able to reduce the amount of cDC1s in vivo. cDC1s may mediate secondary damage of the disease through CD8^+^ T cells, so we detected infiltrating CD8^+^ T cells (CD3^+^CD8^+^) in the spinal cord by flow cytometry. The results showed that CD8^+^ T cells were significantly reduced after Qu addition compared with the SCI group (Fig. 2E). At the same time, the reduced IFN-γ content in the spinal cord tissues of the SCI + Qu treatment group further confirmed the reduced inflammatory response in the injured spinal cord (Fig. 2F).

### cDC1s in LNs surrounding the spinal cord contribute to CD8^+^ T cell infiltration

A previous study demonstrated that the interaction between T cells and DCs in LNs is critical for the initiation of an adaptive immune response (Bousso 2008). Therefore, we collected dcLN and LLN near the spinal cord tissues of sham or SCI group mice for flow cytometry. Compared with the sham group, the proportions of cDC1s (CD11C^+^I-A/I-E^+^XCR1^+^) and proliferating CD8^+^ T cells (CD3^+^CD8^+^Ki67^+^) in dcLN and LLN of SCI group mice were significantly increased (Figure S1A and S1B). We also found that the amount of CD8^+^ T cells (CD8^+^) in dcLN and LLN of SCI mice was decreased after depletion of cDC1s (Figure S1C and S1D), suggesting that CD8^+^ T cell expansion in LNs was regulated by cDC1s.

**Fig. 3:**
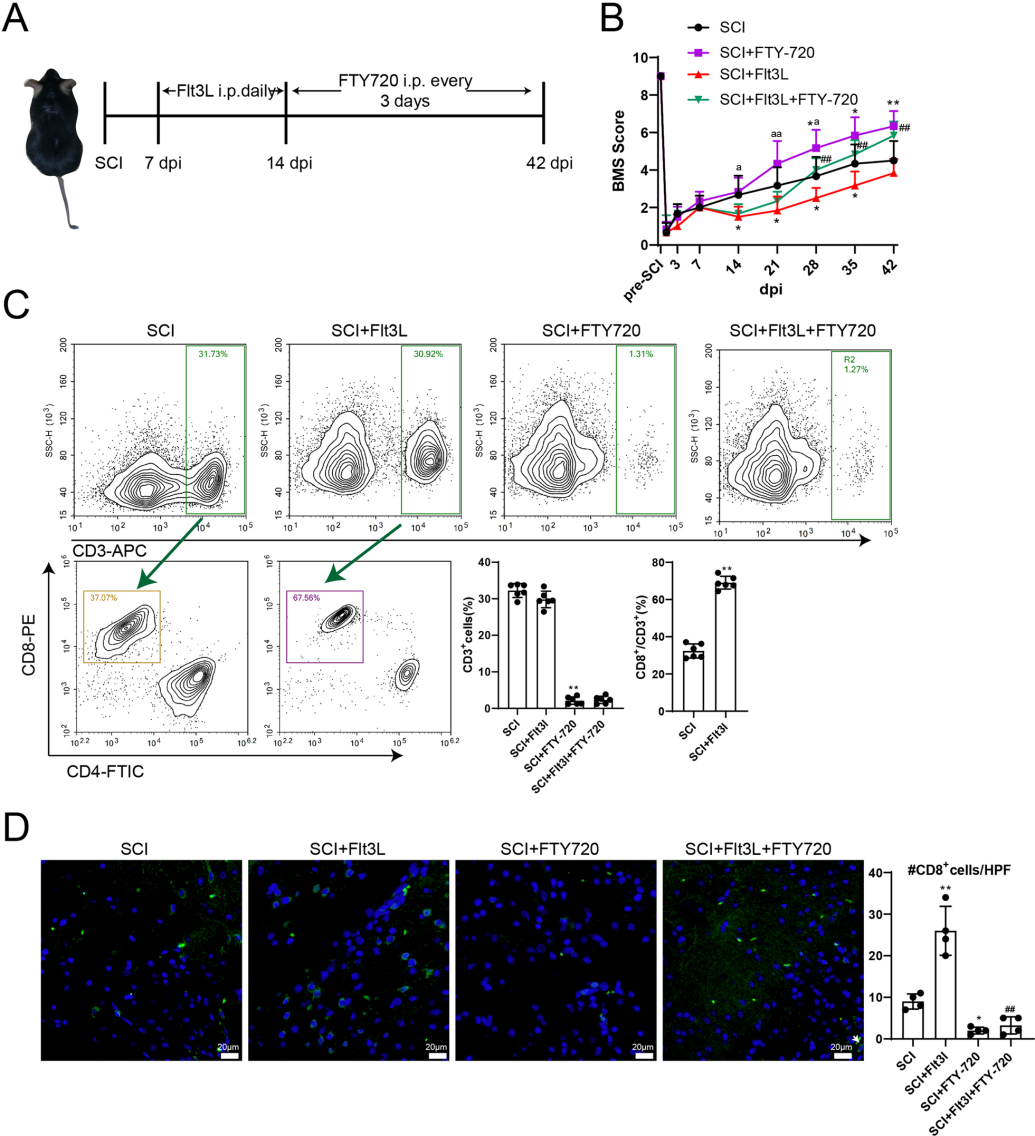
cDC1s in LNs surrounding the spinal cord contribute to the infiltration of CD8^+^ T cells. **A** Mice were grouped into SCI, SCI + FTY-720, SCI + Flt3L, SCI + Flt3L + FTY-720 (*n* = 6/group). Flt3L was injected intraperitoneally every day starting at 7 dpi, and FTY-720 was injected intraperitoneally every 3 days starting at 14 dpi. **B** BMS score detection was performed. The difference was calculated by unpaired two-tailed T-test. *P < 0.05, **P < 0.01 *vs.* SCI group; ^##^*P* < 0.01 *vs.* SCI + Flt3L group; ^a^*P* < 0.05, ^aa^P < 0.01 *vs.* SCI + Flt3L + FTY-720 group. **C** Blood T-lymphocyte subsets (CD3^+^) were detected by flow cytometry. The difference was calculated by one-way ANOVA with Tukey’s post-hoc. **P < 0.01 *vs.* SCI group. *N* = 6. The proportion of CD8^+^ T cells (CD3^+^CD8^+^) in spinal cord tissues was determined by flow cytometry. CD3^+^ T cells were circled with CD3 gate, and further CD3^+^CD8^+^ T cells were analyzed. The difference was calculated by unpaired two-tailed T-test. **P < 0.01 *vs.* SCI group. *N* = 6. **D** The changes of CD8^+^ T cells in spinal cord tissues were detected by IF assay. The difference was calculated by one-way ANOVA with Tukey’s post-hoc. *P < 0.05, **P < 0.01 *vs.* SCI group; ^##^*P* < 0.01 *vs.* SCI + Flt3L group. *N* = 4. *dpi*: days post-injury

To further confirm the effect of cDC1s priming of T cells in LNs on SCI disease, Flt3L was injected intraperitoneally daily starting at 7 dpi to expand cDC1s. As an immunosuppressive drug, FTY-720 blocks the output of T lymphocytes from lymphoid tissues, thereby reducing the distribution of lymphocytes in the blood (Salmon et al. 2016). FTY-720 was injected intraperitoneally every 3 days starting at 14 dpi to prevent T cell egress from LNs (Fig. 3A). The BMS score of SCI + Flt3L mice decreased from 14 dpi, but FTY-720 could significantly restore the BMS score of SCI mice from 28 dpi compared to SCI or SCI + Flt3L groups (Fig. 3B). Compared to the SCI + Flt3L + FTY-720 group, mice in the SCI + FTY-720 group had higher BMS scores from 14 dpi (Fig. 3B). Flow cytometric detection showed that CD3^+^ cells in the blood of mice in the SCI + FTY-720 group were significantly decreased, and the proportion of CD3^+^CD8^+^ T cells was increased in the SCI + Flt3L group (Fig. 3C). Furthermore, we examined the changes in CD8^+^ T cells in spinal cord tissues and found that the addition of Flt3L increased CD8^+^ T cells, whereas FTY-720 decreased the number of CD8^+^ T cells (Fig. 3D). These results suggest that CD8^+^ T cells in LNs may be the main source of CD8^+^ T cell infiltration in injured spinal cord tissues. Since cDC1s in LNs is particularly important for the expansion of CD8^+^ T cells, identifying the cause of increased cDC1s in LNs may serve as a target for SCI treatment.

### Excess non-lymphoid tissue cDC1s in LNs of SCI mice are due to migration of cDC1s from the spinal cord

**Fig. 4:**
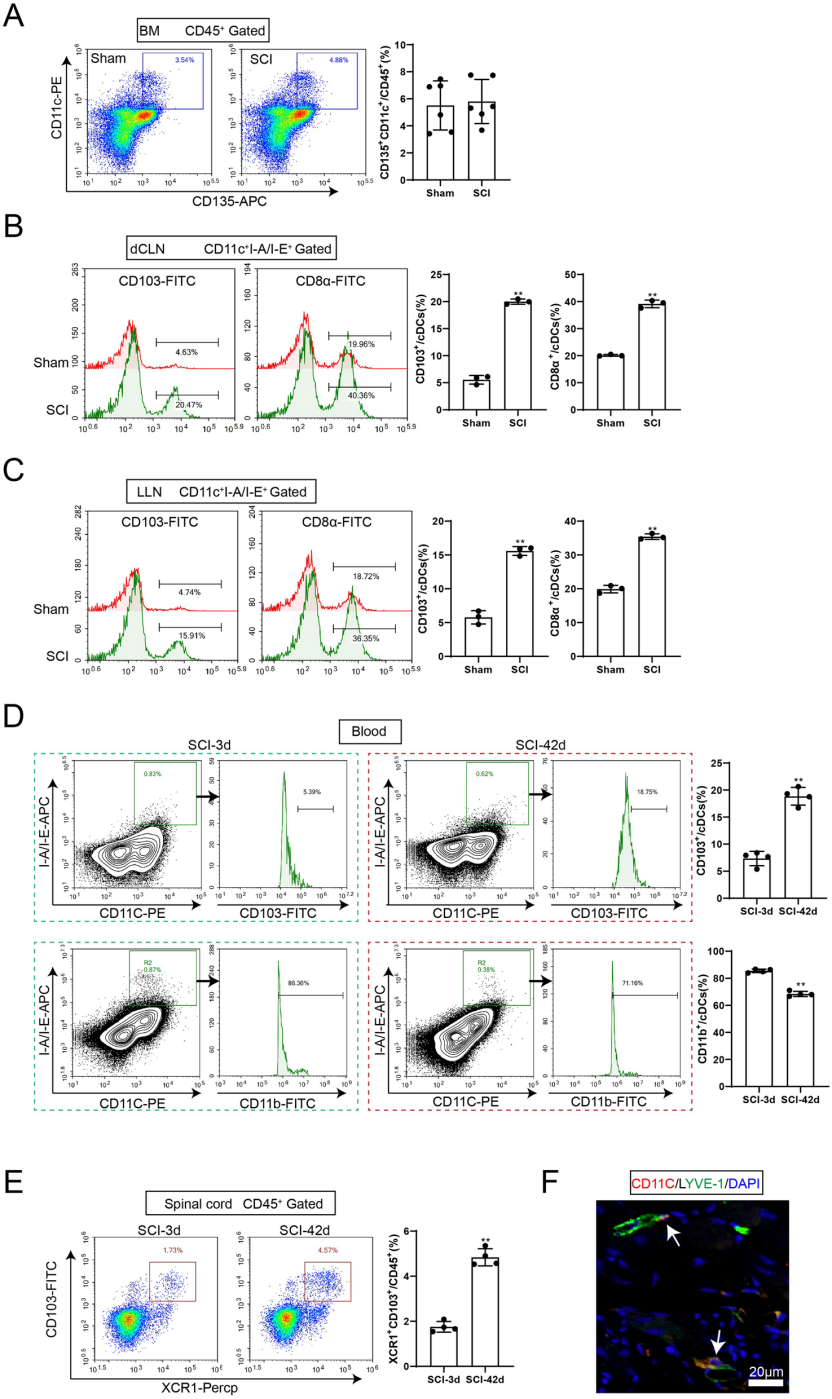
Excess cDC1s in non-lymphoid tissues in LNs of SCI mice is due to migration of cDC1s from the spinal cord. **A** Leukocyte (CD45^+^) and pre-DC subsets in bone marrow of sham and SCI groups were detected by flow cytometry. Immune cells were circled by CD45^+^ and murine pre-DCs were further detected by CD135^+^CD11c^+^. The difference was calculated by unpaired two-tailed T-test. *N* = 6. **B–C** Non-lymphoid tissue cDC1 (CD11C^+^I-A/I-E^+^CD103^+^) proportion and lymphoid-resident cDC1 (CD11C^+^I-A/I-E^+^CD8α^+^) subsets in dcLN and LLN of sham and SCI groups were detected by flow cytometry. The difference was calculated by unpaired two-tailed T-test. **P < 0.01 *vs.* SCI group. *N* = 3. **D** cDC1s (CD11C^+^I-A/I-E^+^CD103^+^) and cDC2s (CD11C^+^I-A/I-E^+^CD11b^+^) subsets in the blood of SCI-3 dpi and SCI-42 dpi groups were detected by flow cytometry. The difference was calculated by unpaired two-tailed T-test. **P < 0.01 *vs.* SCI group. *N* = 6. **E** cDC1 subsets (CD45^+^XCR1^+^CD103^+^) in spinal cord tissues of SCI-3 dpi and SCI-42 dpi groups were detected by flow cytometry. The difference was calculated by unpaired two-tailed T-test. **P < 0.01 *vs.* SCI group. *N* = 6. **F** The positive expressions of CD11C and LYVE-1 in the spinal cord tissues of SCI group mice were detected by IF assay. *BM*: bone marrow; *dcLN*: deep cervical lymph nodes; *LLN*: lumbar lymph nodes

Next, we investigated the source of the excess cDC1s in the LNs of SCI mice. It has been suggested that cDCs originate from the myeloid hematopoietic cascade, where pre-DCs migrate from the bone marrow through the blood to peripheral tissues (including LNs) and differentiate locally into cDC1s or cDC2s (Deczkowska et al. 2021; Cook et al. 2018). The proportion of pre-DCs subsets was determined after isolation of mouse bone marrow. There was no significant difference in the number of leukocytes (CD45^+^) and the proportion of pre-DCs (CD135^+^CD11c^+^CD45^+^) in the bone marrow between the sham and SCI groups (Fig. 4A and S2B), suggesting that the increase in cDC1s may be due to their enhanced differentiation and proliferation in the tissues.

In general, there are two sources of cDC1 in LNs, lymphoid-resident cDC1 and non-lymphoid tissue cDC1, which migrate to LNs through the lymphatic vessels (Merad et al. 2013). Further flow cytometry showed that the proportion of non-lymphoid tissue cDC1 (CD11C^+^I-A/I-E^+^CD103^+^) and lymphoid-resident cDC1 (CD11C^+^I-A/I-E^+^CD8α^+^) was significantly increased in the dcLN and LLN of SCI mice at 42 dpi (Fig. 4B and C). The proportion of cDC1s (CD11C^+^I-A/I-E^+^CD103^+^) was increased in the blood of SCI mice at 42 dpi, while the proportion of cDC2s (CD11C^+^I-A/I-E^+^CD11b^+^) was decreased (Fig. 4D). In addition, the proportion of cDC1s (CD45^+^CD103^+^XCR1^+^) also significantly increased in the spinal cord tissue of SCI mice (Fig. 4E).

DCs have been shown to migrate from inflamed or injured peripheral tissues through lymphatic vessels to the nearest draining LNs, which in turn present foreign antigens to naive T cells (Martín-Fontecha et al. 2009). Therefore, we examined the presence of DCs near lymphatic vessels in spinal cord tissues of SCI mice, and IF assay showed that LYVE-1 and CD11C were co-localized (Fig. 4F). Taken together, these results suggest that increased cDC1s in spinal cord tissues are associated with migration to surrounding LNs and antigen presentation.

### LN-FSCs support the maturation of pre-DCs into lymphoid-resident cDC1s

**Fig. 5:**
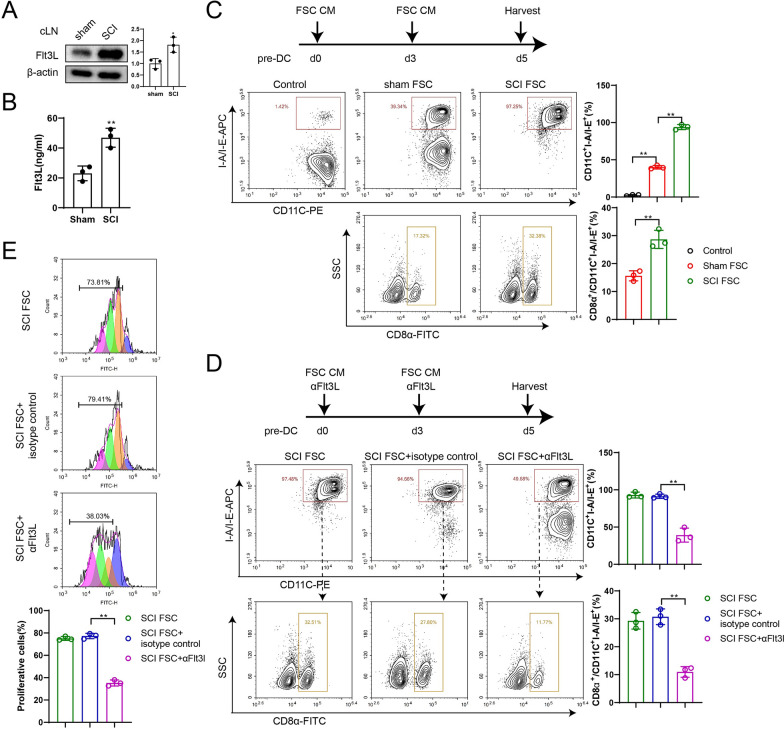
LN-FSCs support the maturation of pre-DCs into lymphoid-resident cDC1s **A** Western blot assay was used to detect Flt3L protein expression in cLN of sham and SCI group mice. The difference was calculated by unpaired two-tailed T-test. *P < 0.05 *vs.* sham group. *N* = 3. **B** LN-FSCs were isolated from sham and SCI group mice. ELISA was used to detect IFN-γ levels. The difference was calculated by unpaired two-tailed T-test. **P < 0.01 *vs.* sham group. *N* = 3. **C** FSC CM were co-cultured with pre-DCs for 5d. Lymphoid-resident cDC1 subsets (CD11C^+^I-A/I-E^+^CD8α^+^) were detected by flow cytometry. The difference was calculated by one-way ANOVA with Tukey’s post-hoc test. **P < 0.01 *vs.* control or sham FSC group. *N* = 3. **D** SCI FSC CM with Flt3L blocking antibody were co-cultured with pre-DCs for 5d. Lymphoid-resident cDC1 subsets (CD11C^+^I-A/I-E^+^CD8α^+^) were detected by flow cytometry. The difference was calculated by one-way ANOVA with Tukey’s post-hoc test. **P < 0.01 *vs.* SCI FSC or SCI FSC + isotype control group. *N* = 3. **E** Lymphoid-resident cDC1 subsets (CD11C^+^I-A/I-E^+^CD8α^+^) from each group were co-cultured with CFSE-labeled CD8^+^ T cells. CD8^+^ T proliferation was assessed by flow cytometry. The multicolored peaks from right to left are CD8^+^ T cells of generation P0, P1, P2 and P3. The proportion of P1–P3 generation cells was counted as cells with proliferative capacity. **P < 0.01. *cLN*: cervical lymph node; *CM*: conditioned medium

We next investigated the reasons for the increase in resident cDC1s within the LNs. High protein expression of Flt3L was detected in LNs of SCI mice by Western blotting (Fig. 5A), and higher levels of Flt3L were also found in the supernatant of LN FSCs in the SCI group (Fig. 5B), suggesting that SCs in LNs may promote the differentiation of cDC1s by secreting Flt3L. After LN FSCs were isolated from mice in the sham and SCI groups, the corresponding CM were co-cultured with pre-DCs for 5 d and then detected by flow cytometry. Figure 5C showed that DC subsets (CD11C^+^I-A/I-E^+^) were increased after pre-DCs were cultured in FSC CM, and the proportion of lymphoid-resident cDC1s (CD11C^+^I-A/I-E^+^CD8α^+^) was more significantly increased in the SCI FSC group.

To further confirm the effect of Flt3L on the differentiation of lymphoid-resident cDC1s, αFlt3L antibody was added to block Flt3L when pre-DCs were co-cultured with SCI FSC CM. The results showed that the proportion of lymphoid-resident cDC1s (CD11C^+^I-A/I-E^+^CD8α^+^) was significantly reduced after the addition of αFlt3L (Fig. 5D). This result indicates that Flt3L in SCI FSC CM affects the lineage differentiation of lymphoid-resident cDC1s. CD8α^+^ cDC1s (CD11C^+^I-A/I-E^+^CD8α^+^) induced by SCI FSC CM or SCI FSC + αFlt3L CM were co-cultured with CFSE-labeled CD8^+^ T cells, and the proliferation ability of CD8^+^ T cells was detected by flow cytometry. The results showed that the proliferation-promoting effect of SCI FSC CM on CD8^+^ T cells was greatly attenuated after Flt3L inhibition (Fig. 5E).

### IFN-γ promotes Flt3L production by LN-FSCs through Janus kinase (JAK)/STAT signaling pathway

**Fig. 6:**
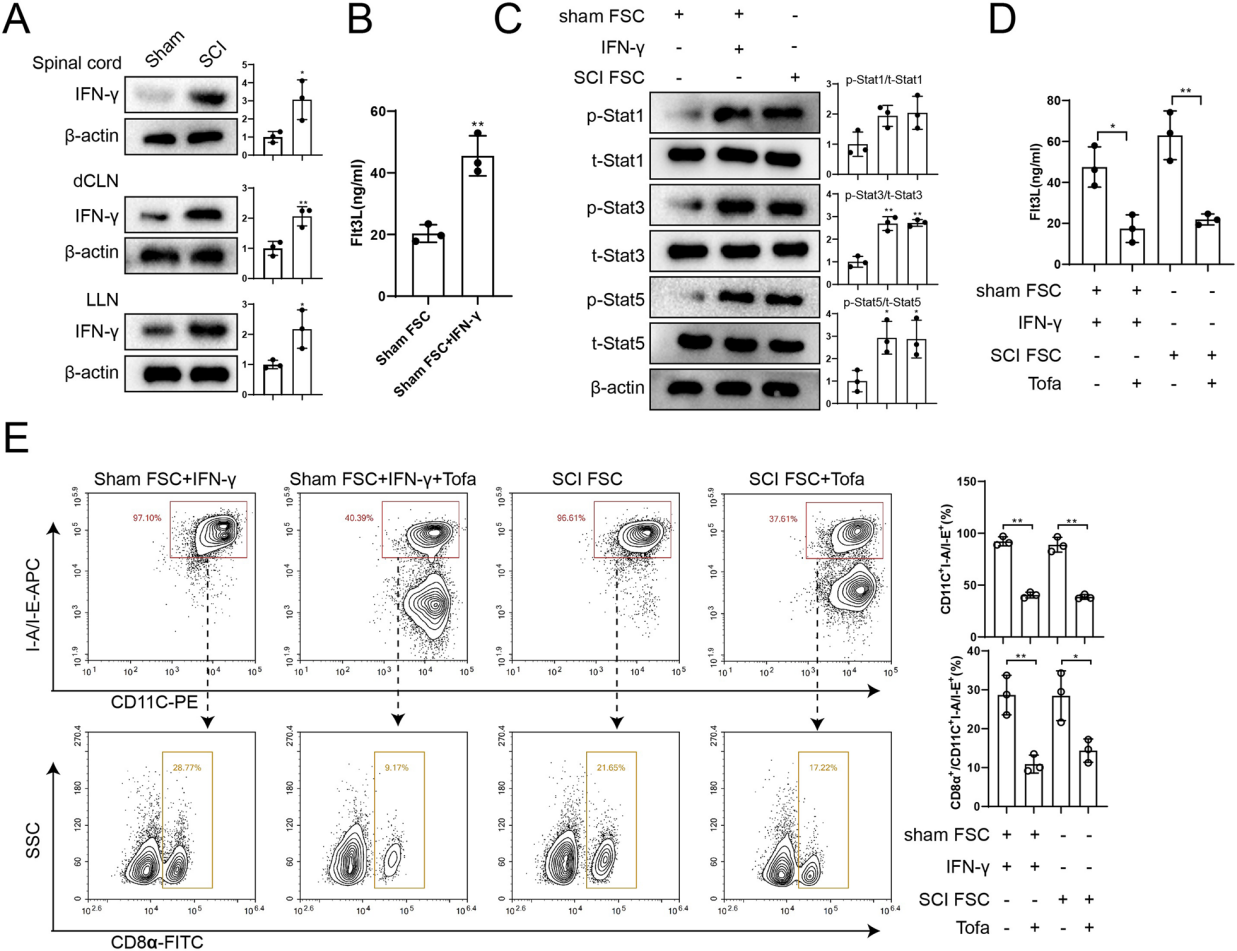
IFN-γ promoted the production of Flt3L by LN-FSCs through the JAK/STAT pathway. **A** Western blot assay was used to detect IFN-γ protein expression in spinal cord tissues, dcLN, and LLN of sham and SCI group mice. The difference was calculated by unpaired two-tailed T-test. *P < 0.05, **P < 0.01 vs sham group. *N* = 3. **B** ELISA was used to detect Flt3L levels in sham FSC and sham FSC + IFN-γ groups. The difference was calculated by unpaired two-tailed T-test. **P < 0.01 *vs.* sham FSC group. *N* = 3. **C** Western blot assay was used to detect p-Stat1/3/5 and t-Stat1/3/5 protein expression in sham FSC, sham FSC + IFN-γ, and SCI FSC groups. The difference was calculated by one-way ANOVA with Tukey’s post-hoc test. *P < 0.05, **P < 0.01 *vs.* sham FSC group. **D** ELISA was used to detect Flt3L levels in the sham FSC + IFN-γ, sham FSC + IFN-γ + Tofa, SCI FSC, and SCI FSC + Tofa groups. The difference was calculated by one-way ANOVA with Tukey’s post-hoc test. *P < 0.05, **P < 0.01 *vs.* sham FSC + IFN-γ or SCI FSC groups. **E** FSC CM were co-cultured with pre-DCs for 5 days, and grouped into sham FSC + IFN-γ, sham FSC + IFN-γ + Tofa, SCI FSC, and SCI FSC + Tofa groups. Lymphoid-resident cDC1s (CD11C^+^I-A/I-E^+^CD8α^+^) were detected by flow cytometry. The difference was calculated by one-way ANOVA with Tukey’s post-hoc test. *P < 0.05, **P < 0.01 *vs.* sham FSC + IFN-γ or SCI FSC groups. *dcLN*: deep cervical lymph nodes; *LLN*: lumbar lymph nodes; *Tofa*: tofacitinib

Both spinal cord tissues and LNs of SCI mice contained high protein expression of IFN-γ (Fig. 6A). The level of Flt3L in FSCs of the sham group was increased after IFN-γ stimulation (Fig. 6B). Yuan et al*.* found that the JAK/STAT pathway was involved in IFN-γ-induced upregulation of Flt3L expression in umbilical cord-derived mesenchymal stem cells (Yuan et al. 2019). Therefore, we detected p-Stat1/3/5 and t-Stat1/3/5 protein expressions in the sham FSC, sham FSC + IFN-γ, and SCI FSC groups and found that the expressions of JAK/STAT pathway-related proteins were enhanced (Fig. 6C).

Next, the pan-JAK inhibitor tofacitinib (Tofa) was used to further verify whether the JAK/STAT pathway regulates Flt3L expression in FSCs. Figure 6D showed that the amount of Flt3L in sham FSC + IFN-γ was reduced after Tofa treatment, and the amount of Flt3L in the SCI FSC group was also significantly reduced. Next, CM of FSCs with or without Tofa treatment were co-cultured with pre-DCs and then used to examine the ability of pre-DCs to differentiate into lymphoid-resident cDC1s (CD11C^+^I-A/I-E^+^CD8α^+^). Compared with the sham FSC + IFN-γ group or the SCI FSC group, the proportion of lymphoid-resident cDC1s (CD11C^+^I-A/I-E^+^CD8α^+^) was significantly decreased after inhibition of the JAK/STAT pathway (Fig. 6E). Therefore, we reasoned that IFN-γ in LNs could promote Flt3L production by FSCs via the JAK/STAT pathway.

## Discussion

The adaptive immune response occurs in the body to fight against exogenous antigens or antigens produced by autoimmunity. After SCI, the functional adaptive immunity in the spinal cord will be activated, but the post-traumatic induced autoimmunity will exacerbate the injury (Jones 2014). Therefore, inhibiting the secondary SCI caused by adaptive immunity is very important to improve the prognosis of SCI patients. DCs are important cells that mediate adaptive immune responses. By recognizing danger signals released by the body, cDC1s take up antigens and cross-present them to CD8^+^ T lymphocytes (Sanmamed and Chen 2018). This study is the first to investigate the effect of cDC1s on neuroinflammation after SCI by promoting the expansion of CD8^+^ T cells. Clinical peripheral blood samples were collected from SCI patients and it was found that the proportion of CD141^+^ cDC1s was significantly higher than that of healthy controls. XCR1 is a relatively conserved molecule on the surface of DCs and is expressed on the surface of mouse, human and porcine cDC1s (Auray et al. 2016; Crozat et al. 2010). XCR1 binds to soluble or endogenous antigens and is presented to CD8^+^ T cells via MHCII to initiate cellular immune responses (Crozat et al. 2010). The positive expression of XCR1 or MHCII was detected by IF assay in the animal experiments of the present study, and the results showed that the cDC1s content was increased in the injured spinal cord of SCI mice. Clinical examination and in vivo experiments showed that the proportion of cDC1s in the injured spinal cord was significantly increased, and further depletion of cDC1s in mice reduced CD8^+^ T cell infiltration after SCI injury.

cDC1s are professional antigen-presenting cells, and the accumulation of resident cDC1s in the tumor improved CD8^+^ T cell expansion, which is critical for the induction of protective CD8^+^ T cell responses (Salmon et al. 2016). In this study, after intraperitoneal injection of Flt3L into SCI mice to expand CD103^+^ cDC1s, we found that the expansion of CD8^+^ T cells was increased and the motor function of the mice was more damaged. Of note, it has been reported that implantation of DCs into the injured spinal cord after SCI can activate neurogenesis and promote recovery of motor function (Yaguchi et al. 2009; Hayashi et al. 2009). However, the specific subtypes of DCs were not specified in these studies, and the effects of different subtypes of DCs were different, which does not contradict the results of the present study. When SCI mice were injected with the immunosuppressive drug FTY-720 to prevent T cells from exiting the LNs, the levels of CD8^+^ T cells in the blood and spinal cord tissues and the BMS scores were restored, suggesting that CD8^+^ T cells in the LNs may be the main source of CD8^+^ T cell infiltration at the injured spinal cord tissues.

The present study is the first to investigate the molecular mechanism of increased cDC1s in LNs of SCI mice. On the one hand, CD103^+^ cDC1s in spinal cord tissues migrated to LNs, *i.e.*, migratory cDC1s. On the other hand, FSCs in LNs promoted the differentiation of pe-DCs into CD8α^+^ cDC1s by secreting Flt3L, *i.e.*, resident cDC1s. In recent years, researchers have successfully induced and differentiated mouse bone marrow-derived DCs by using cytokines such as granulocyte–macrophage colony-stimulating factor, interleukin-4, Flt3L, and so on (Helft et al. 2015; Mayer et al. 2014). Flt3L is an essential growth factor for cDC1s development and is produced by stromal cells (SCs) (Chen et al. 2023; Cueto and Sancho 2021). In vivo injection of Flt3L into mice has been shown to promote the differentiation and survival of cDC1s (Lin et al. 2020). In addition, SCs play an important role in the formation and development of secondary lymphoid organs (Wiechers et al. 2022). In this study, we detected high Flt3L expression in LNs and isolated LN-FSCs of SCI mice, and the differentiation of pre-DCs into resident CD8α^+^ cDC1s in LNs was promoted by Flt3L secreted from FSCs. In addition, the effect of resident cDC1s induced by SCI FSCs on CD8^+^ T-cell proliferation was greatly reduced after inhibition of Flt3L in vitro, further confirming that cDC1s promoted CD8^+^ T-cell proliferation in LNs around the SCI spinal cord.

The pro-inflammatory cytokine IFN-γ is mainly involved in autoimmune and inflammatory responses, and the main pathway of its activation is JAK/STAT (Nan et al. 2018). JAK is a key factor upstream of the STAT pathway, and the JAK/STAT pathway is a key signaling pathway for immune system activation. Various cytokines (including IFN-γ) bind to receptors to activate trans-phosphorylation of JAK, which then recruits and catalyzes the phosphorylation of STAT. The phosphorylated STAT forms a dimer in the nucleus to initiate transcription of target genes and then induce expression of various cytokines (Guo et al. 2023). Studies have reported that the JAK/STAT pathway can affect cell survival, growth, and differentiation, and plays an important role in the pathological changes of SCI (Guo et al. 2023). In vitro, high levels of IFN-γ were detected in spinal cord tissues and LNs of SCI mice. We also found that JAK inhibitor reversed IFN-γ-induced enhancement of Flt3L production by FSCs as well as directed maturation of cDC1s in LNs. In the future, JAK inhibitors may be developed to inhibit adaptive immunity after SCI.

**Fig. 7:**
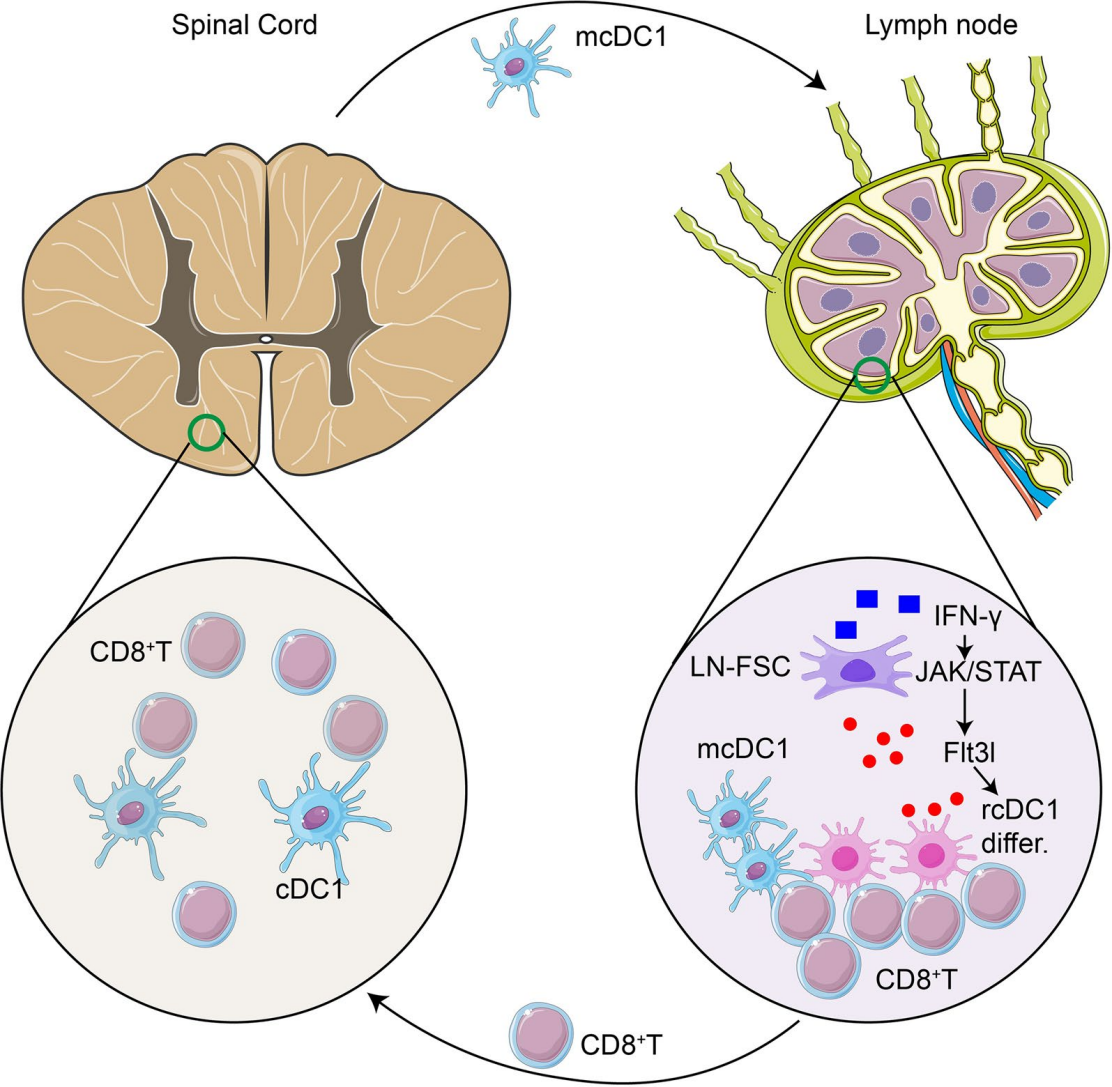
The increase of cDC1s in the peri-spinal lymph nodes after SCI was partly derived from mcDC1s (CD103^+^XCR1^+^ cDCs) at the damaged site of the spinal cord, and partly due to the stimulation of the intracellular JAK/STAT pathway by IFN-γ in the peri-spinal lymph nodes to promote the secretion of FLT3L, thereby promoting the differentiation of resident cDC1s (CD8α^+^ cDCs). The increase in cDC1s in the lymph nodes promotes the expansion of CD8^+^ T cells, resulting in an increase in the number of CD8^+^ T cells infiltrating the damaged tissues of the spinal cord, leading to a chronic, long-term adaptive immune response. *mcDC1*: migratory cDC1; *rcDC1*: resident cDC1s; *LN-FSC*: lymph node fibroblastic stromal cells

In conclusion, cDC1s mediate adaptive immune responses through CD8^+^ T cells in SCI, and migratory and resident cDC1s promote CD8^+^ T cell expansion in LN (Fig. 7). This study may provide a theoretical basis for the treatment of SCI by intervening in the immune inflammatory response, which has practical significance.

## Supplementary Information

Additional file 1: Figure 1. (A-B) Mice were divided into sham (*n*=6) and SCI (*n*=6) groups. The proportion of cDC1s (CD11C^+^I-A/I-E^+^XCR1^+^) or proliferating CD8^+^ T cells (CD3^+^CD8^+^Ki67^+^) in dCLN and LLN was determined by flow cytometry. The difference was calculated by unpaired two-tailed T-test. **P < 0.01 *vs. *SCI group. (C-D) Mice were divided into SCI (*n*=6) and SCI+Qu (*n*=6) groups. The proportion of CD8^+^ T cells in dCLN or LLN was determined by flow cytometry. The difference was calculated by unpaired two-tailed T-test. **P < 0.01 *vs. *SCI group. dcLN: deep cervical lymph nodes; LLN: lumbar lymph nodes; Qu: Quizartinib.

Additional file 2: Figure 2. (A) Mice were divided into SCI (*n*=6) and SCI+Qu (*n*=6) groups. Qu administration was started on day 14 after surgery. The cDC population was expressed as CD11C and I-A/I-E (MHCII) double positive, and XCR1^+^ represents XCR1^+^ cDCs. (B) Leukocytes (CD45^+^) and pre-DCs subsets in the bone marrow of sham and SCI groups were detected by flow cytometry. The immune cells were circled by CD45^+^ and murine pre-DCs were further detected by CD135^+^CD11c^+^.

## Data Availability

The datasets used and/or analyzed during the current study are available from the corresponding author on reasonable request.
