# Supplementary figures and images for: Conventional type 1 dendritic cells in the lymph nodes aggravate neuroinflammation after spinal cord injury by promoting CD8+ T cell expansion
Source: Mol Med. 2025 Feb 3;31:37. doi: 10.1186/s10020-024-01059-4 (PMC11789313; doi:10.1186/s10020-024-01059-4)

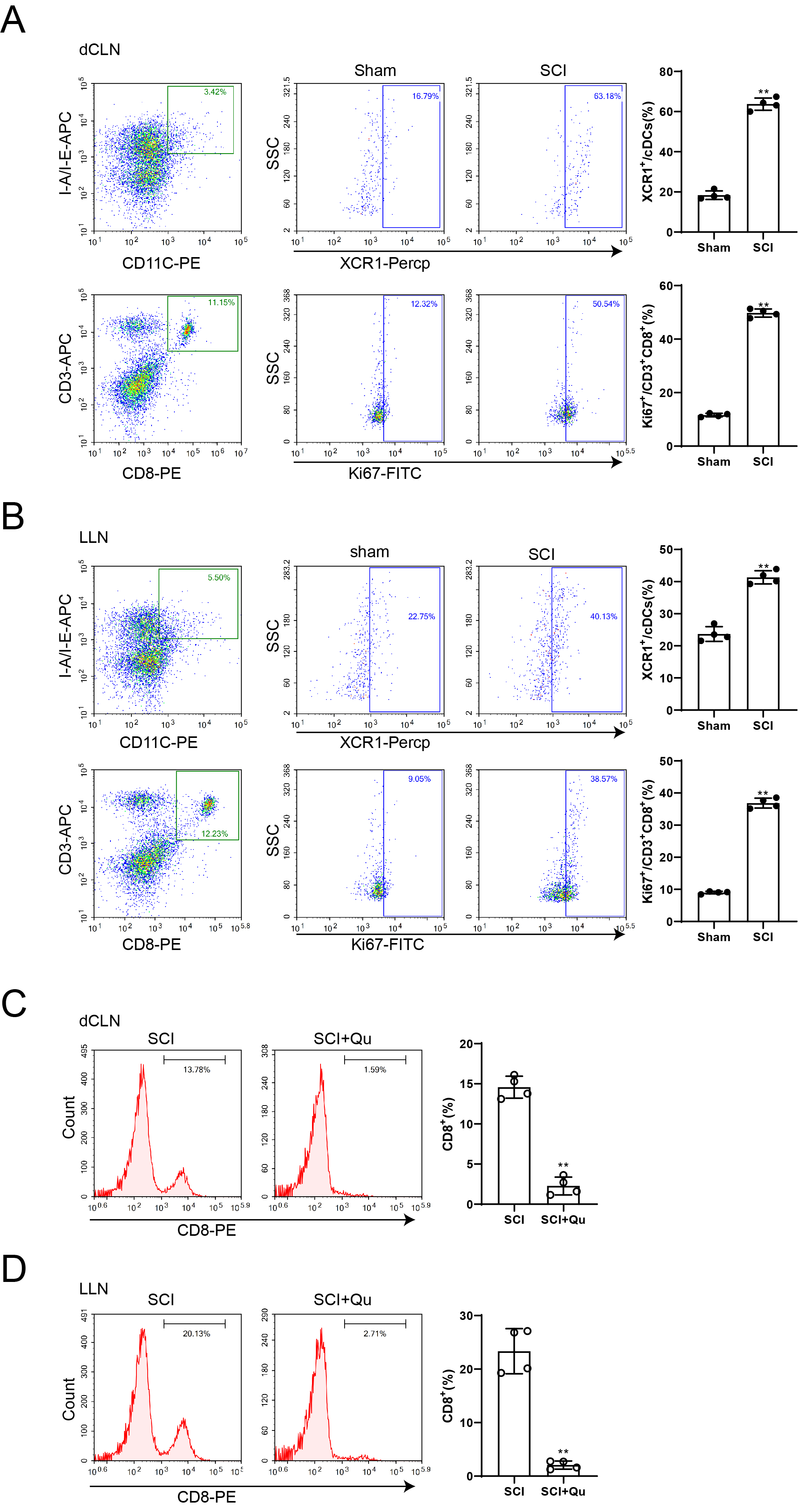

Supplement: Supplementary file 1 — Additional file 1: Figure 1. (A-B) Mice were divided into sham (n=6) and SCI (n=6) groups. The proportion of cDC1s (CD11C+I-A/I-E+XCR1+) or proliferating CD8+ T cells (CD3+CD8+Ki67+) in dCLN and LLN was determined by flow cytometry. The difference was calculated by unpaired two-tailed T-test. **P < 0.01 vs. SCI group. (C-D) Mice were divided into SCI (n=6) and SCI+Qu (n=6) groups. The proportion of CD8+ T cells in dCLN or LLN was determined by flow cytometry. The difference was calculated by unpaired two-tailed T-test. **P < 0.01 vs. SCI group. dcLN: deep cervical lymph nodes; LLN: lumbar lymph nodes; Qu: Quizartinib. [file 10020_2024_1059_MOESM1_ESM.jpg]

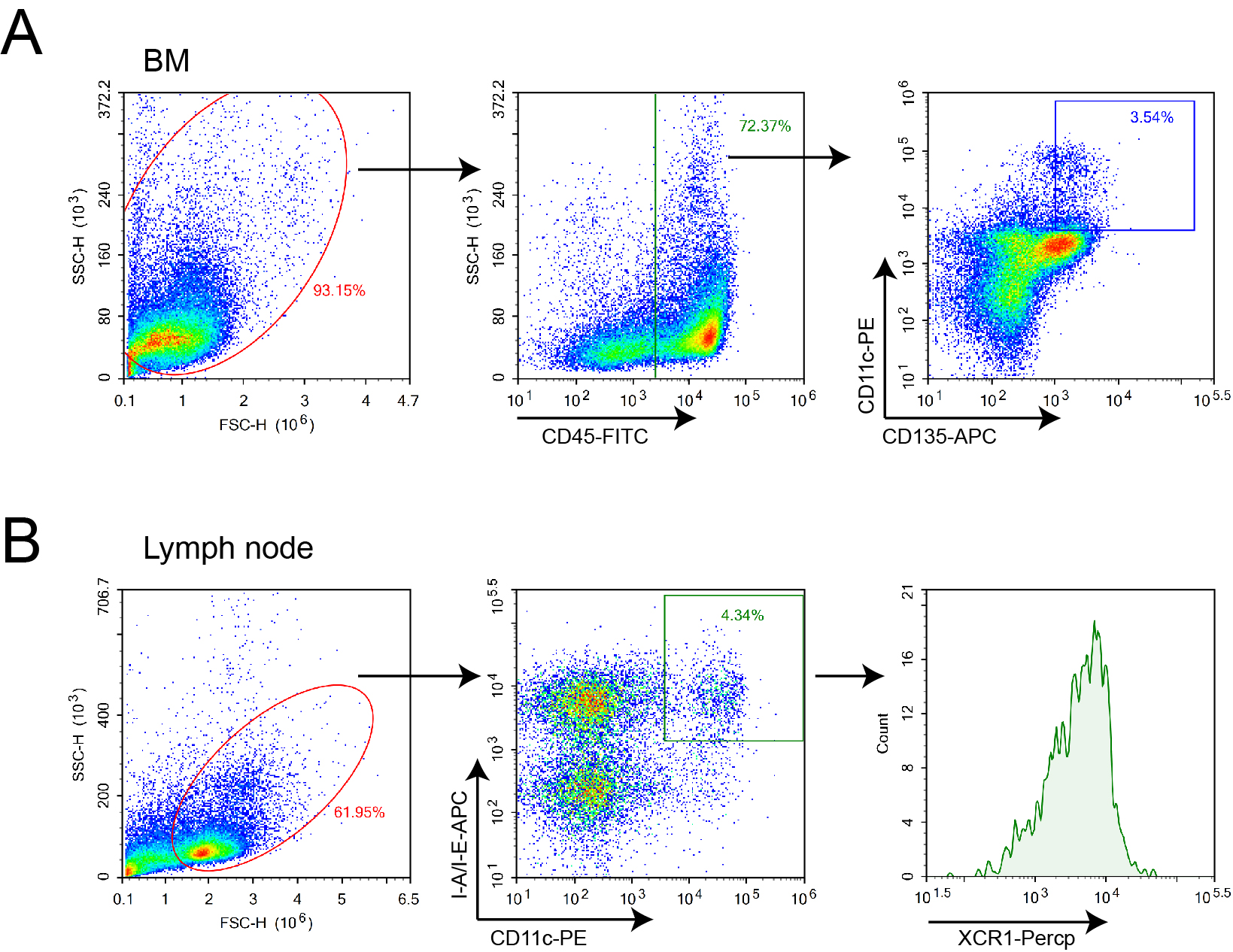

Supplement: Supplementary file 2 — Additional file 2: Figure 2. (A) Mice were divided into SCI (n=6) and SCI+Qu (n=6) groups. Qu administration was started on day 14 after surgery. The cDC population was expressed as CD11C and I-A/I-E (MHCII) double positive, and XCR1+ represents XCR1+ cDCs. (B) Leukocytes (CD45+) and pre-DCs subsets in the bone marrow of sham and SCI groups were detected by flow cytometry. The immune cells were circled by CD45+ and murine pre-DCs were further detected by CD135+CD11c+. [file 10020_2024_1059_MOESM2_ESM.jpg]
